# Sebaceous gland carcinoma of the eyelid

**DOI:** 10.4103/0974-620X.71885

**Published:** 2010

**Authors:** Upender K. Wali, Abdullah Al-Mujaini

**Affiliations:** Department of Ophthalmology, College of Medicine and Health Science, Sultan Qaboos University, Muscat, Sultanate Oman

**Keywords:** Moh’s, nodular, pagetoid, sebaceous gland carcinoma

## Abstract

Sebaceous gland carcinoma, commonly arises in the periocular area, is an uncommon condition. It represents 1–5.5% of eyelid malignancies and is considered to be the third most common eyelid malignancy after basal cell and squamous cell carcinomas, although few reports placed this tumor as second most common after basal cell carcinoma. It usually affects elderly women and characterized by high rate of local recurrence, regional, and distant metastases. A delay in diagnosis, which can be attributed primarily to ability of this tumor to masquerade as more benign conditions, often leads to inappropriate management with increased morbidity and mortality rates. In this study, the authors discuss key elements of the primary disease and therapeutic options available to treat such devastating problem.

## Introduction

Sebaceous gland tumor of the eyelids may arise from the meibomian glands, glands of Zeis or glands associated with the caruncle.[1] They are included in the list of tumors of the epidermal appendages, so-called adnexal skin structures. Sebaceous gland carcinoma (SGC) might be the second most common lid malignancy after basal cell carcinoma (BCC). Its multifocal origin and pagetoid spread give it an unique place among eyelid malignancies.

Sebaceous glands are located in the periocular skin, caruncle, and eyebrow skin follicles. The tumor is a very rare, slow growing, and commonly found in elderly population with female predisposition. Mean age at diagnosis is mid-sixties; however, the tumor has been reported in children as young as 3.5 years old.[2] It is rare in Caucasians and common in oriental Asiatics. The reported incidence of SGC varies from 0.5 to 5% of all lid carcinomas in USA and 28% in China.[3,4] SGC most commonly arises from the meibomian glands anterior to the gray line, occasionally from the glands of Zeis or Moll, and from sebaceous glands in caruncle; however, the cell of origin may not be certain in 50–60% of cases.[5] In contrast to basal cell carcinoma (BCC) or squamous cell carcinoma (SCC), SGC is two to three times more common in upper eyelid due to more number of meibomian glands there.[3,6] Five percent cases may have simultaneous involvement of both eyelids due to intraepithelial spread and/or spontaneous development of multiple primaries.

On one hand it can mimic as benign lesion as blepharoconjunctivitis, whereas on the other extreme it can have widespread local and fatal distant metastases. Immunohistochemistry, molecular biology, and electron microscopy have greatly improved the diagnosis, management, and prognosis of SGCs overall. Surgery, chemotherapy, and radiotherapy all contribute to the treatment of SGC.

The literature was reviewed using online searches of PubMed (1985–2010), OVID, and Google Scholar. The search strategy included MeSH and natural language terms using the keywords mentioned. Reference lists in retrieved articles, and textbooks, were also searched for relevant references.

## Relevant Anatomy

With its two parts; the epithelial or epidermis and the cutaneous or dermis, the skin of the eyelid is the thinnest in the body. Anatomically, the eyelid can be divided into two lamellae. The anterior lamella contains skin, orbicularis muscle, eyelashes, and their follicles whereas the posterior lamella has mucocutaneous junction, meibomian gland orifices, and tarsal plate.

The dermis contains sebaceous glands which are found in the tarsal plate, caruncle, and eye brow. There are approximately 25 such oil producing sebaceous glands in the upper tarsal plate and 20 in the lower. Glands of Zeis are small, modified, sebaceous secreting glands that open into the hair follicles at the base of the eyelashes.

## Pathophysiology

Two important features differentiate sebaceous carcinoma from other periocular malignancies. First, unlike single origin of other tumors, sebaceous carcinoma appears to arise from multifocal origins. Second, unlike radial spread of basal cell and SCCs, SGC tends to spread superficially in a pattern known as pagetoid spread.[7,8] This spread may lead to the erroneous histologic diagnosis of epithelial dysplasia or carcinoma *in situ*.

**Figure 1:**
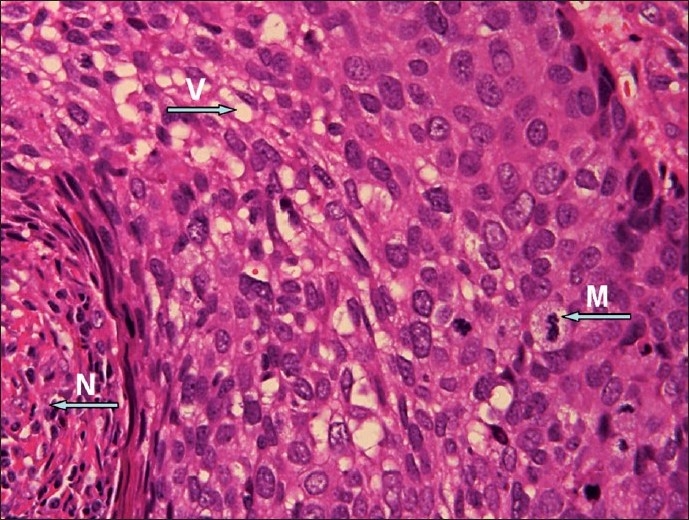
Malignant cells with vaculated cytoplasm (V), mitotic figures (M), and areas of necrosis (N) (H & E, ×60)

Immunohistopathology shows that the cells occur in irregular lobular masses with distinctive invasiveness. The cytoplasm is pale, foamy, and vacuolated [Figure 1]. This feature of foamy cytoplasm is seen only in sebaceous carcinoma. The nuclei are hyperchromatic, and the cells stain positive for lipid such as oil red O stain. The ultrastructural features of SGC include desmosoes, tonofilaments, and intracytoplasmic nonmembrane bound lipid. Occasionally bizarre and atypical tripolar mitotic cells may be seen. Immunohistochemical reaction aids in the diagnosis of SGC. Molecular biology of SGC suggests dysplasia if there is no expression of p53 or invasiveness, if there is hyperexpression of p53.

## Clinical Features

SGC has a tendency to invade the periocular region. Upper eyelid is most commonly involved followed by the lower eyelid and the caruncle.[9]

It bears no characteristic clinical appearance, but pagetoid infiltration of conjunctival epithelium or skin epidermis is a hallmark of this tumor. It is one of the most dangerous eyelid tumors due to; a) the tumor masquerading as inflammatory conditions such as blepharo-conjunctivitis, chalazion or superior limbic keratoconjunctivitis,[10–12] or as other ocular tumors like BCC or SCC, with a result that the correct diagnosis is often delayed until metastasis has occurred, b) the incidence of metastasis is high (41%),[3,5,7] c) delineation of tumor margins, even with excellent paraffin-embedded sections is difficult due to either intraepithelial pagetoid spread and/or multicentric pattern,[5,6,8,13–15] and d) the histological diagnosis may be incorrect if lipid stains (oil-red-O) are not used on properly prepared tissues.

In general, there are two main pathological presentations of the SGC - nodular and spreading.

## The Nodular Form

**Figure 2:**
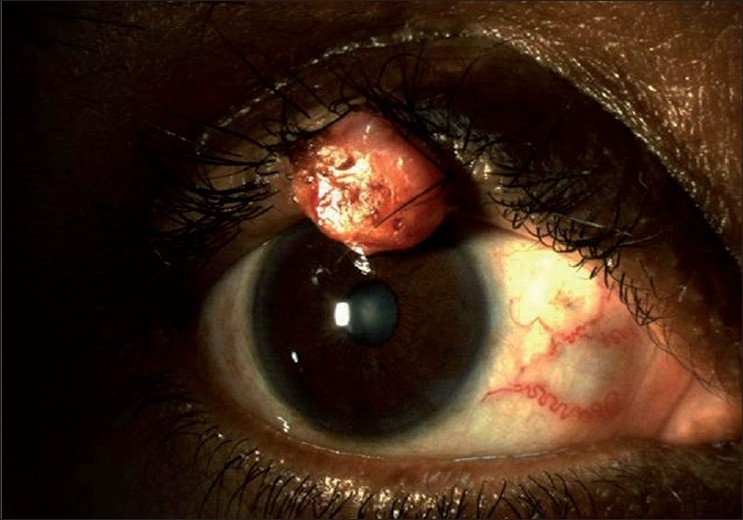
Clinical presentation showing large mass of the right upper eyelid

In over 50% cases, SGC may present as a pseudochalazion or a chronic blepharoconjunctivitis.[10–12,16,17] The nodular form is a discrete, hard, immobile nodule commonly located in upper tarsal plate [Figure 2]. It is yellowish due to lipid. Any chalazion of unusual consistency or its recurrence after incision and curettage more than three times should undergo full thickness resection and histological examination. Occasionally, it may have a multicentric origin.

The spreading variety of SGC occurs in pagetoid form with diffuse intraepithelial infiltration of the lid skin.[8,11,13] This causes diffuse thickening of lid margin, loss of eyelashes, and resembles chronic blepharoconjunctivitis. The pagetoid spread may involve both eyelids and conjunctival epithelium.[13]

An unusual variant of SGC may present as Muir–Torre syndrome which is characterized by squamous differentiation of the tumor, multiple sebaceous adenomas or carcinomas in the skin, and visceral malignancies.

SGC is important to know because it is a notorious diagnostic pitfall for the clinician as well as the pathologist. It is the pagetoid spread or the dermal infiltration of the tumor that may confuse both the clinician and the pathologist. The clinician may misdiagnose SGC as blepharoconjunctivitis, and the pathologist as dysplasia. Accurate diagnosis and treatment of SGC are important because this tumor is the most aggressive of the epithelial tumors of the eyelid. Any unilateral blepharoconjunctivitis with loss of eyelashes, thickening of the lid margin and that fails to respond to the treatment should be biopsied, so should be any suspicious area of conjunctiva or the limbus.

Sebaceous gland hyperplasia constitutes 8% of caruncular growths. SGC of caruncle may involve mucous membrane graft of the defect after excision of the tumor.

The systemic extension of the tumor can occur by continuous growth, lymphatic spread, or hematogenous spread. The most common sites of spread are orbit, preauricular and/or submandibulr nodes, and parotid gland. The less common sites of extension include cervical nodes, lung, pleura, liver, brain, pericardium, lips, ethmoid sinus, or skull.[3,5,6,18,19]

## Diagnosis

Diagnosis is frequently difficult due to two main reasons; (1) in early stages the external signs are quite subtle, resembling a benign lesion such as a chalazion or chronic blepharoconjunctivitis and (2) the presence of yellowish material within the tumor gives it resemblance to SCC.

Thus, due to high index of suspicion, it becomes mandatory to include SGC in the differential diagnosis of most eyelid masses and recurrent, nonresponding eyelid inflammatory conditions.

A delay in clinical diagnosis of SGC can be attributed primarily to its ability to masquerade as more common benign eyelid conditions. This often leads to delayed management with increased morbidity and mortality rates.[20] The actual reported sebaceous carcinoma-related mortality is about 6%.[7]

## Differential Diagnosis

The list for SGC includes; congenital sebaceous gland hyperplasia which is common on face or scalp or acquired sebaceous gland hyperplasia which is common on face or forehead. Adenoma sebaceum of Pringle is another diagnosis to consider. It is found in tuberous sclerosis and commonly located in the nasolabial fold and cheek areas. Sebaceous adenoma is common on the eyebrows and eyelids.

SGC is included in the group of simulating lid lesions (inclusion cyst, papilloma, senile keratosis, keratoacanthoma, benign keratosis, dermoid cyst, and amyloidosis).

Occasionally, SGC may diffusely infiltrate conjunctival epithelium. This may resemble lesions such as chronic blepharoconjunctivitis, superior limbal keratoconjunctivitis or cicatricial pemphigoid, and BCC. The tumor may mimick chronic inflammation and even grow bacteria on repeated cultures but does not improve with antibiotics. Conjunctival scrapping may reveal the underlying SGC pathology. Due to such mimicking, SGC is one of the forms of masquerade syndromes. Diffuse intraepithelial sebaceous carcinoma closely resembles SGC in its rarity and indolent course; however, the former is not an obvious primary tumor of the eyelid and may not require exenteration. Papilloma simulates SGC and sometimes may be differentiated only by histology.

## Treatment

SGCs are often inadequately treated at first intervention. Different treatment modalities include local excision, orbital exenteration, radical neck dissection, radiation, or chemotherapy depending on the stage of the tumor at the time of presentation. Wide excision at an early stage is important. Prior to surgical excision, it is important to examine the patient carefully for evidence of pagetoid spread or multicentric origin by double eversion of the eyelids, and any conjunctival alteration such as telangiectasia, papillary change, or a mass. In such an instance, conjunctival punch biopsies should be taken in addition to surgical resection of the lid lesion.[21]

**Figure 3:**
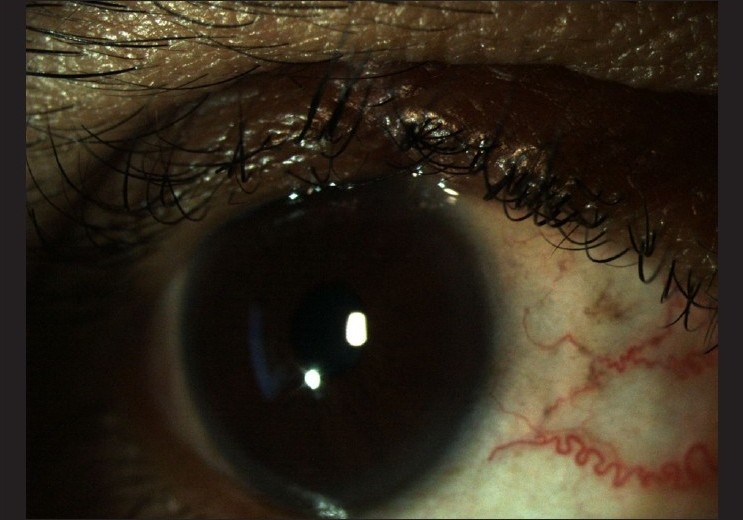
Two weeks after surgical resection of the tumor and reconstruction of the eyelid

Surgical treatment may range from a local excision to orbital exenteration [Figure 3]. Radical surgical excision with frozen section control by either a standard method or Moh’s micrographic surgery is the most common and effective method of treatment. The wound edges should be approximated as far as possible. Approximately, 30% of SGCs recur after resection.[18,22]

The advantages of Moh’s micrographic surgery, in comparison to a conventional excision of nonmelanotic skin cancer are; (1) definitive margin excision and (2) minimal loss of surrounding normal tissue.[23]

Few patients with regional nodal metastasis remain alive for long time, and radical neck dissection for isolated cervical node disease is often indicated.[6] Local nodal disease without distant metastasis is treated by radical neck dissection.[7,18,19]

Topical mitomycin C has been tried for pagetoid invasion of the conjunctiva by eyelid SGC.[24] Cryotherapy is a useful adjunct to surgery in epibulbar and pagetoid extension of SGC, sparing exenteration.

Radiotherapy is usually avoided or may even be contraindicated because of its adverse side effects such as subsequent conjunctival keratinization leading to dry eye, lid atrophy, skin necrosis, lash loss, lid telangiectasia, ectropion, epiphora, keratopathy, cataract,[25] higher recurrence rate compared to surgery, does not allow histological confirmation of tumor classification and eradication and finally recurrences following radiotherapy are difficult to treat surgically because of poor healing of irradiated tissues. Most serious radiation-related complications occur in large tumors of the upper lid.

The reported results of proton electron irradiation in SGC are not excellent.[22,26] Radiotherapy as a primary form of therapy in SGC of the eyelid has variable results.[7,26,27] Radiation is mainly reserved for patients who are not candidates for surgical procedures due to advanced age or disease, for palliation in widespread disease, and for patients who refuse exenteration for advanced local disease. In some cases, wide-field irradiation has either cured the tumor or produced sufficient shrinkage to allow surgical removal of the residual mass. As a bias, surgery has been recommended for SGC confined to the lid, and to limit the use of radiation to diffuse tumors with orbital or bone involvement. In advanced poorly differentiated tumors, cure is not affected by either radiotherapy or orbital exenteration.

## Prognosis [Table 1]

The overall mortality rate is 5–10% because of frequent difficulties, mistakes in diagnosis, and delay in the treatment. The mortality from metastasis may go up to 25%. The adverse prognostic features include involvement of upper or both eyelid and tumor size of 10 mm or more. Others include duration of symptoms more than 6 months (mortality 38%), poorly differentiated tumors, infiltration into blood vessels and/or lymphatics, orbital extension, multicentric origin, and finally pagetoid spread. Tumors less than 6 mm have excellent prognosis. Prognosis of SGC arising from glands of Zeis is more favorable.

**Table 1:** Prognosis of sebaceous gland carcinoma (SGC) based on clinicopathological tumor morphology[28]

*Well differentiated SGC*	*Moderately differentiated SGC*	*Poorly differentiated SGC*
Resemble histogenetic architecture of the sebaceous gland lobule (peripheral stem cell layer and central sebaceous cells)	Retain lobular pattern. Positive staining reaction for fat	Difficult to see or absent positive staining reaction for fat

It goes beyond doubt that SGC is a great mimicker. On one hand, it mimics as simple a clinical condition as blepharitis, on the other hand it may turn out to be a fatal metastatic tumor.
